# Massive Burns: Retrospective Analysis of Changes in Outcomes Indicators Across 18 Years

**DOI:** 10.1093/jbcr/irab072

**Published:** 2021-04-22

**Authors:** Joachim N Meuli, Olivier Pantet, Mette M Berger, Laurent Waselle, Wassim Raffoul

**Affiliations:** 1 Department of Plastic and Hand Surgery, Lausanne University Hospital, Switzerland; 2 Department of Adult Intensive Care Medicine and Burns, Lausanne University Hospital, Switzerland; 3 Cell Production Center, Lausanne University Hospital, Switzerland

## Abstract

The treatment and management of massive burns, defined as burns affecting at least 50% of total body surface area (TBSA), have considerably changed since the 1990s. This study aimed at analyzing if the length of intensive care unit (ICU) stay, the success of skin grafting operations, and the mortality changed in the past 18 years. Between 2000 and 2018, 77 patients were admitted for massive burns to the ICU of a university hospital. Transfers and early care withdrawal precluded inclusion for 38 patients, leaving 39 for analysis. Study variables were year of admission, demographics, burn characteristics, critical care treatment (fluid resuscitation, ventilation, and nutrition), and surgical therapy. Association between outcomes and year of admission was assessed through correlation and logistic regression analysis. Potential confounders were assessed through stepwise linear regression. Patients’ characteristics were stable over time with a median age of 36 (25.0–48.0) years, burns 65% (55.0–83.0) TBSA, and deep burns 55% (50.0–68.0) TBSA. Length of ICU stay remained stable at 0.97 (0.6–1.5) days/%TBSA. Mortality was stable as well. Energy and carbohydrate delivery decreased in parallel with the number of infectious episodes per patient. The number of operations was stable, but the take rate of skin grafts increased significantly. The multivariate analysis retained year of admission, weight, the total number of infections, daily lipid intakes, and fluid resuscitation as independent predicting variables.

## BACKGROUND

Burns are a frequent trauma with an estimated incidence of 9 million cases per year worldwide and a prevalence of 90 million cases in 2017.^1^ The vast majority are benign cases as the area affected and/or the depth of the injury are limited. The incidence of severe burns, usually defined as burns more than 20% of total body surface area (TBSA) in adults and/or by the need of specialized burn intensive care units (ICUs) treatment,^2,3^ is not precisely known, but estimates range from 160,000 to 2.3 million cases per year worldwide.^4^ Massive burns are a more imprecise term, with a threshold ranging from more than 35 to more than 60% TBSA depending on the authors, and are estimated to represent 8 to 10% of all admissions in burns centers,^5^ that is, 12,000 to 230,000 cases per year worldwide. Massive burns have generally occurred in the context of wars or industrial accidents and have become less frequent in the last decade thanks to significant improvements in workplace safety, fire prevention, and regulations, foremost in high-income and upper-middle-income countries.^4,6^ They, however, still occur and remain the most expensive and complex trauma hospitals have to deal with. Charges are routinely higher than half a million U.S. dollars per case,^7,8^ patients often remain hospitalized for several months and in-hospital mortality up to 54% has been reported.^9^

## OBJECTIVE

In this review of the massively burned patients treated at Lausanne University Hospital burn ICU, we investigated whether the length of ICU (LICU) stay, the success of skin grafting operations, and the mortality changed between 2000 and 2018. During this period, incremental changes to nutritional support protocols were introduced in order to decrease energy delivery and carbohydrate delivery.^10^ Targets for the initial resuscitation volume were adapted as well, becoming more restrictive and surgical procedures were updated with the standardization of cultured epithelial autografts (CEAs) use. We hypothesized that the optimization of resuscitation procedures and of nutrition therapy combined with the use of novel surgical techniques improved outcomes.

## MATERIALS AND METHODS

### Study Design

This retrospective cohort study was designed as the analysis of a subset of a previously published study’s population.^10^ Approval from the local ethics committee was obtained at the time for the entire population (CER-VD 2018-02268). The study was registered on clinicaltrials.gov (NCT04480944.)

### Setting

Data of patients admitted to the burn ICU of the Lausanne University Hospital, an 1100-beds quaternary care hospital in Switzerland, between January 1, 2000 and December 31, 2018 were retrieved. Analysis and redaction were completed in 2020.

### Patients

All adult patients (>18 years old) with burns at least 50% TBSA admitted in the burn ICU of the Lausanne University Hospital were included in this study. The limit of more than 50% TBSA was chosen as an approximate average of previously used limits that ranged from more than 35% to more than 60% TBSA.^11–14^ Patients admitted with another primary diagnosis than burn (eg, necrotizing fasciitis, gangrene, and toxic epidermal necrolysis) were excluded as their management differs from classical burns. Patients referred from or to another ICU were excluded in order to maintain control over variables and patients in which active withdrawal of care was decided within 48 hours of admission were excluded, as previously done by other authors.^15^ Therapy withdrawal was a consensus decision of the medical and surgical teams, based on the severity of injuries, comorbidities, permanent disability perspective, and patient’s age. Patients with first-degree burns were excluded because they do not need surgical treatment, as in other similar studies.^16^

### Variables

The following cohort characteristics were retrieved: patients’ age, sex and weight, type and date of injury, overall TBSA burned, TBSA burned to deep second-degree and third-degree (deep burns TBSA), occurrence of inhalation injury, SAPS2 score, and modified Baux score.

Treatments and complications that occurred during the ICU stay were also retrieved: fluid resuscitation received during the first 48 hours (initial resuscitation), nutritional intakes (average daily energy, lipid, carbohydrates, and protein intakes related to body weight), the total number of infectious episodes, number of cutaneous infections, number of surgical interventions, total area grafted, type and number of cultured skin autografts used, length of mechanical ventilation, and length of stay in the ICU. Fluid resuscitation was calculated over 48 hours because the resuscitation protocols for massive burns at our institution recommend a calculated resuscitation volume for this period of time (4 ml/kg/%TBSA for the first 24 h and 2 ml/kg/%TBSA for 24–48 h). Afterward, volume infusions are based on monitoring values. The primary outcome of the study was the length of stay in the ICU, corrected by the TBSA burned (LICU/%TBSA). This approach permits us to take into account the fact that injury characteristics are the most important predictors of lengths of stay.^2,5,17^ The main secondary outcome was the success of skin grafting operations, assessed through both the raw number of operations and the ratio between the TBSA grafted and the deep burns TBSA. This ratio was calculated as such: sum of TBSA grafted at each operation during ICU stay/estimated deep burn TBSA. The use of a ratio is not ideal but direct skin graft take rates evaluations have been shown to be poorly standardized,^18^ and similar ratios have been used before to evaluate CEAs take rates.^19,20^

Other secondary outcomes were the daily nutritional intakes (energy, proteins, lipids, and carbohydrates) as well as the overall mortality.

### Data Sources/Measurement

Population’s characteristics, treatments received, and occurrence of complications were collected from the clinical information system of the ICU (MetaVision^®^ 5.46.44; iMDSoft, Tel Aviv, Israel) as well as from discharge letters and operative reports in the hospital electronic medical records (Soarian^®^; Cerner, North Kansas City, USA). Nutritional values were reported per kilogram of pre-admission weight. Infectious episodes were defined according to the International Sepsis Forum Definition of Infection in the ICU Consensus Conference^21^ and the American Burn Association Consensus Conference to Define Sepsis and Infection in Burns.^22^ No distinction was made between nosocomial and community-acquired infections and the severity of infection was not assessed. The site of infection, however, was specified in order to identify skin infections according to Greenhalgh et al.^22^

The overall TBSA burned, the area defined as deep burns TBSA, and the area grafted were assessed by plastic and reconstructive surgery residents or attending physicians who were present at admission or at the time of operation. If the evaluation of the TBSA affected or of the burn depth changed during the ICU stay, the latest value was chosen. Data regarding cultured skin autografts were retrieved from the hospital’s Cell Production Center (CPC) manufacturing records. This center is an independent structure within the hospital that took over the production of CEAs from the plastic and reconstructive surgery’s cutaneous grafts laboratory (which started clinical use in 1985) and later expanded the range of available products to cultured sprayed keratinocytes and bilayered cultured dermo-epithelial autografts. The percentage of TBSA covered by cultured autografts was calculated using manufacturing data of the CPC (area in m^2^) divided by the TBSA of the patients calculated according to Mosteller.^23^

Several individuals who were part of the ICU team and the plastic and reconstructive surgery team performed data collection over time. All data were controlled and re-assessed by the first author. Doubtful situations were reviewed with a senior author.

### Quantitative Variables

Continuous variables are presented as medians and interquartile ranges (25–75), while discrete data are presented as full numbers or percentage. Normality was tested using Shapiro–Wilk test given the limited number of observations (n = 39).

### Statistical Analysis

Correlation between the year of admission and the primary outcome (LICU/%TBSA) as well as between the year of admission and the main secondary outcome (ratio between TBSA grafted and deep burns TBSA) was assessed using Kendall’s rank correlation test or Pearson’s correlation test depending on normality. In both cases, the analysis was performed on a restricted dataset after exclusion of all patients who died during their ICU stay to prevent biasing the result with artificially low values due to death. Previously published literature on this topic followed the same approach.^24,25^ Difference over time in mortality was tested using logistic regression analysis.

A stepwise linear regression analysis was performed in case of statistically significant differences in outcomes (either primary or main secondary) in order to identify non-cofounding predicting variables.

There was no missing data. All analyses were performed using the R Project for Statistical Computing version 3.6.2. A *P* value less than .05 was considered significant. Variables that did not show a significant change over time were defined as stable.

## RESULTS

### Patients

**Figure 1.**
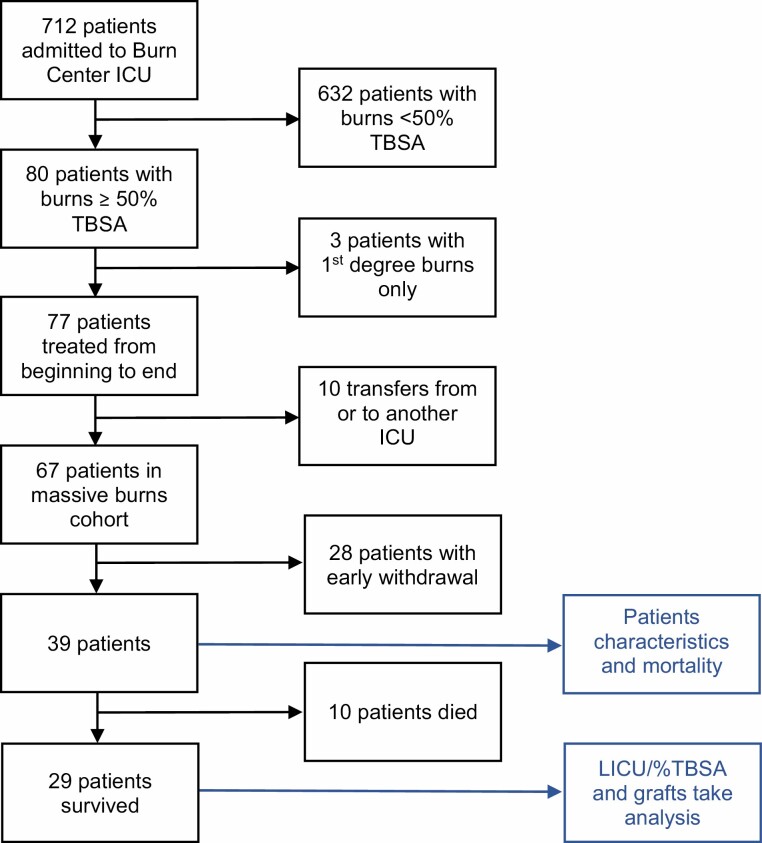
Flow diagram.

Altogether 712 patients were admitted to the burn ICU in the selected timeframe: 80 patients (11.2%) with at least 50% TBSA burned but 3 had only first-degree burns and were therefore excluded. Ten patients were transferred from or to another ICU and excluded. In 28 patients, active care was withdrawn within 48 hours of admission. All of the remaining 39 patients were followed up from admission until discharge from the ICU and were included (Figure 1).

### Descriptive Data

Patients’ characteristics are presented in Table 1: They were stable over time. Median age was 36 years (25.0–47.5) with a predominance of males (33 males vs 6 females). Median TBSA burned was 65% (55.0–82.5). Non-survivors and patients with early withdrawal shared characteristics: they were older (*P* < .05), more severely burned (*P* < .05), and had higher Baux scores (*P* < .05) than the survivor group.

**Table 1.** Patients’ characteristics, medians (IQR)

Characteristic	Total Population	Survived	Died	*P* Value
	n = 39	n = 29	n = 10	
Age (years)	36.0 (25.0–48.0)	28.0 (25.0–39.0)	58.0 (48.5–59.0)	<.001
Female (n (%))	6 (15.4)	5 (17.2)	1 (10)	.969
Weight (kg)	74.40 (68.0–83.2)	72.7 (67.9–83.0)	78.0 (71.2–85.4)	.421
TBSA (%)	65.0 (55.0–83.0)	60.0 (53.0–72.0)	84.0 (66.3–90.0)	.007
TBSA deep burns (%)	55.0 (50.0–68.0)	50.0 (45.0–60.0)	71.0 (63.3–82.3)	.001
Inhalation injury (n (%))	25 (64.1)	19 (65.5)	6 (60.0)	1.000
SAPS2 score	36.0 (32.0–45.0)	35.00 (32.0–40.0)	51.0 (44.0–59.0)	<.001
Modified Baux score	113.0 (101.0–130.0)	111.0 (98.0–116.0)	148.0 (129.0–157.3)	<.001

*IQR*, interquartile range; *TBSA*, total body surface area.

**Figure 2.**
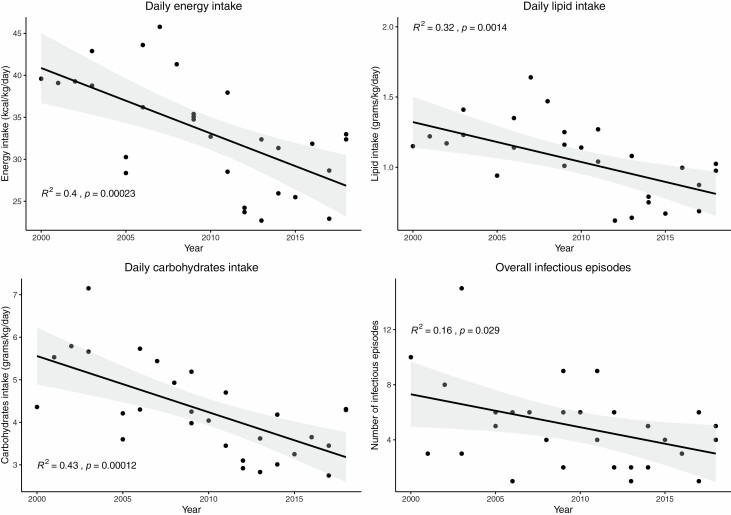
Evolution in nutritional therapy and infectious complications over time showing a significant reduction in total daily energy, lipid, and carbohydrate delivery, as well as a decrease in the total number of infectious complications per patient.

Table 2 displays the treatments received during the ICU stay: median 48-hour fluid resuscitation volume was 26 liters (21.4–28.7), representing 5. 8 ml/kg/%TBSA (4.8–6.4) and did not change significantly over time. Median length of ventilation was stable with 27.8 days (19.0–52.5). Patients underwent a median of six operations, representing a median TBSA grafted of 52.0% (27.0–87.5), both numbers stable over time. Only the overall number of infectious episodes and some nutritional values (see outcomes data) decreased (Figure 2).

**Table 2.** Treatments received and in-hospital complications, medians (IQR)

Treatments Received/In-Hospital Complications	Total population	Survived	Died	*P* Value
	n = 39	n = 29	n = 10	
Fluid resuscitation (ml/kg/%TBSA)	5.8 (4.8–6.4)	5.8 (5.1–6.4)	5.9 (3.8–6.3)	.499
Length of mechanical ventilation (days)	27.8 (19.0–52.5)	25.4 (18.5–37.9)	47.4 (20.5–70.1)	.359
Energy intake (kcal/kg/day)	32.1 (28.4–36.1)	32.7 (28.5–38.8)	29.5 (26.6–31.7)	.072
Protein intake (g/kg/day)	1.6 (1.3–1.7)	1.6 (1.4–1.8)	1.6 (1.2–1.7)	.247
Lipid intake (g/kg/day)	1.0 (0.8–1.2)	1.0 (0.9–1.2)	0.9 (0.8–1.0)	.122
Carbohydrates intake (g/kg/day)	4.0 (3.4–4.5)	4.2 (3.5–4.9)	3.7 (3.2–4.0)	.157
Cutaneous infections (n)	1.0 (0.5–2.0)	1.0 (0.0–2.0)	1.5 (1.0–4.5)	.167
Overall infections (n)	5.0 (2.5–6.5)	5.0 (3.0–6.0)	5.0 (2.3–8.8)	.783
Skin grafting surgeries (n)	6.0 (3.0–8.0)	6.0 (4.0–8.0)	4.5 (0.3–7.0)	.324
Length of ICU stay/% TBSA	1.0 (0.6–1.5)	1.0 (0.8–1.5)	0.7 (0.3–1.4)	.247
Ratio TBSA grafted/deep burns TBSA	1.1 (0.7–1.4)	1.1 (0.8–1.5)	0.5 (0.0–1.3)	.115

*IQR*, interquartile range; *TBSA*, total body surface area; *ICU*, intensive care unit.

CEAs were used in 32 patients (82%), covering a median body surface area of 27.9% (9.9–54.1). Cultured dermo-epithelial autografts and sprayed keratinocytes were used only anecdotally in five patients (12%) and three patients (7%), respectively.

### Outcome Data

**Figure 3.**
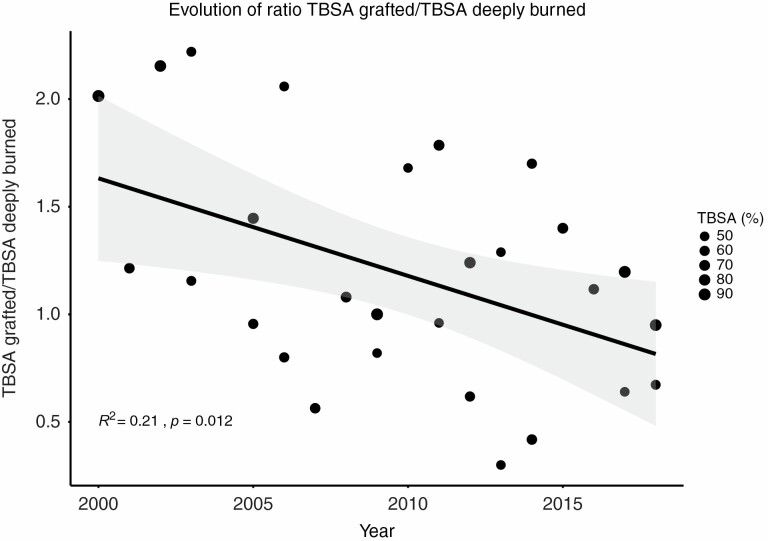
Correlation between year of admission and ratio TBSA grafted/deep burns TBSA.

The LICU/%TBSA did not change over time with a median of 0.97 days (0.64–1.47) for an overall median LICU of 62.0 days (36.0–105.5). The median number of operations during ICU stay remained stable at 6 (4–8), but the ratio between area grafted and area deeply burned decreased over time from 1.56 to 0.96 with a significant correlation (*r* = −0.46, *P* < .05) between this ratio and the year of admission (Figure 3).

Mean daily energy delivery decreased significantly over the years, stabilizing slightly over 30 kcal/kg/day (*P* < .05). Protein delivery increased modestly from 1.3 to 1.6 g/kg/day. The most significant nutritional change was the reduction of carbohydrate delivery from 5.5 to 3.5 g/kg/day (*P* < .05).

The mortality rate was 25.6% over the 18 years period and was not associated with the year of admission.

A stepwise linear regression analysis to assess which variables were non-cofounding predictors of the ratio of TBSA grafted over deep burns TBSA produced a model that included the year of admission, the body weight, the total number of infections, the daily lipid intake, and the initial fluid resuscitation. The carbohydrate intake, despite its significant reduction over time, did not appear in it. This model had an adjusted *R*^2^ value of 0.5141 (*P* < .05).

## DISCUSSION

### LICU Stay per TBSA

The hypothesis that the changes implemented over 18 years affected the LICU stay proved to be wrong. Both the absolute length of stay as well as the value corrected by the TBSA burned remained stable over time. Different models predicting the length of hospital stay^24,26,27^ and LICU stay^25^ for burned patients have been developed, and these models show that patients’ characteristics such as age (≥65 years old), third-degree burns, and inhalation injury are key predictors of a longer stay. These characteristics remained stable over time in this study. The same models show as well that in-hospital acquired complications (infections and respiratory failure), surgical complications (ie, skin graft loss), and nutritional support also have significant effects on the LICU stay.^25,28,29^ In this study, the total number of infectious episodes decreased and the nutritional support changed significantly, which should have affected the length of stay favorably. Moreover, skin grafts take rates improved over time (see secondary outcomes) which should have further contributed to shorter stays.

We, therefore, do not have an obvious explanation for the stability of this outcome over time. One important predictor of LICU stay, the length of mechanical ventilation, remained stable and can partly explain it. Another possible explanation might be the changes in patients’ flow or in available resources locally. Indeed, patients leaving the burn ICU are transferred to intermediate or standard care, and changes in the flow of patients may affect the LICU stay more importantly than changes in treatments. Lastly, a hypothetical improvement in LICU stay could have been attenuated by the fact that shortening this outcome has not been, until now, an objective in our burn ICU. In consequence, the medical and surgical strategies applied in the acute phase aimed primarily at the best recovery, accepting the LICU stay as being a consequence of these decisions. Comparing the LICU stay to the overall length of hospital stay and/or to functional outcomes would provide a valuable insight into the adequateness of this outcome.

### Skin Grafts Take Rate

The number of operations during the ICU stay showed a tendency to decrease over time, but this trend was not significant (*P* = .057). We however observed a significant improvement in the ratio of TBSA grafted/TBSA deeply burned. In the years 2012 to 2018, this ratio reached a mean value of 0.96 ± 0.43 vs 1.59 ± 0.52 in the years 2000 to 2005 (*P* < .05, not shown). This suggests a significant improvement in the take rate of skin grafts, thereby reducing the need to re-graft the same body area several times.

There is limited published literature on factors influencing skin grafts for severely burned patients, but evidence in smaller burns, combined with physiological rationale, shows that thickness of graft, soft tissue bed, shear forces, infections, surgical technique, and nutrition are all major influencing factors.^30–32^ Graft thickness and soft tissue bed remained constant in this study as our burn center standard practice has been to use 0.2 mm skin grafts since before 2000 and tissue bed preparation only changed in the late 2010s when fetal cell bandages began to be used in adults patients after successful trials on children.^33^ Two patients included in this cohort benefited from this new therapy and while further studies are underway to assess the potential benefits, it seems unlikely that this change affected our results.

On the other hand, shear forces, infections, surgical technique, and nutrition all changed to a certain extent during the timeframe of this study and could partly explain the improvement we observed. For example, the systematic use of pre-filled two-component fibrin sealant (ARTISS) in skin grafting operations as from 2010 might have provided an increased resistance to shear forces compared to the previously used glue. The quantity of fibrin sealant used was unfortunately not recorded in our system and could not be traced back to test this hypothesis. The reduction of the number of infections (but not of cutaneous infections) per patient might as well have influenced skin grafts healing. The increased use of a “sandwich technique” in which CEAs are applied over highly (3:1 to 6:1) meshed skin grafts to enhance take rates^34^ while producing aesthetic and functional results similar to smaller meshing ratio used alone^35,36^ might have contributed, but the increase in this technique over the 18 years of our study was not significant (*P* = .19, not shown). Lastly, changes in nutrition have probably affected the skin graft take rates both directly and through infection prevention (see Discussion on nutrition).

Coherently, the stepwise regression analysis performed to determine which factors independently affected this outcome retained the number of infections as well as the lipid intake but not the changes in surgical techniques Table 3. With an adjusted *R*^2^ value of 0.5141 (*P* < .05), this model highlights the importance of yet-to-be-determined variables that were unaccounted for. Those could possibly be found either in planned but unrecorded changes (eg, use of pre-filled fibrin sealant) or unplanned and unrecorded changes (eg, timing of skin grafting operations in regard to infectious episodes).

**Table 3.** Stepwise linear regression analysis

	Coefficient	Standard Error	*t* Value	*P* Value	Tolerance	Variance Inflation Factors
*Variables*						
Year of admission	−0.037	0.017	−2.232	.035	0.610	1.64
Weight (kg)	−0.012	0.007	−1.864	.075	0.525	1.91
Overall infections (n)	0.098	0.024	3.984	.001	0.793	1.26
Daily lipid intake (g/kg)	−0.696	0.395	−1.760	.091	0.427	2.34
Initial resuscitation (ml/kg/%TBSA)	−0.077	0.055	−1.386	.178	0.672	1.49
Dependent variable: ratio TBSA grafted/TBSA deep burns						
Adjusted *R*^2^ 0.5141				0.0004		

*TBSA*, total body surface area.

### Nutrition

Energy prescription in the burn ICU was guided by indirect calorimetry, but the translation of the measured energy expenditure (mEE) values to prescription has evolved over time. In the late 1990s, the standard recommendation was to apply 1.5 × mEE for severely burned patients. The strategy later evolved and decreased to 1.3 × mEE, while in the last decade, the mEE has been applied without any correction factor.

The reduction in carbohydrates delivery, which was very high in the early 2000s, was achieved by changing the feeding products to less glucose-containing solutions and by tightening control over the intravenous delivery of glucose 5% solutions. This came in response to studies showing a maximal oxidation rate of glucose at 5 mg/kg/h,^37^ deleterious effects of hyperglycemia on graft take rates,^38^ and impact on mortality.^39^ The correlation we observed between glucose administration and the total number of infections (*P* < .05, not shown) supports these results. Blood glucose control was introduced in 2001 and proven to be safe.^40^ All combined, these nutritional changes may have contributed to improve the graft take rate. Of note, all the patients included in this study received trace element repletion treatment.^41^

Glutamine administration has been part of our nutrition therapy since 2006,^37^ and this strategy was confirmed with the 2013 European Society for Clinical Nutrition (ESPEN) burn guidelines which recommend including glutamine supplementation. The reduction of carbohydrates is also recommended by ESPEN (maximum of 55% of total energy), and the possible association between energy delivery and skin graft take rates highlighted in this study underlines the importance of these nutritional guidelines in severe burns care.

### Mortality

Given the limited size of the sample, the high heterogeneity of the patients included, and the known limitations of using this endpoint in intensive care,^42^ the failure to show a change in mortality over time is no surprise. Mortality in burned patients has been shown to have reached a plateau in the last decade after a significant reduction between the 1950s and 1980s and might have become too small as a target to achieve.^28,43–45^ The decision to exclude all patients with early withdrawal could have biased our results if withdrawals occurred more frequently at certain periods of time or if the criteria that lead to withdrawal changed over time. Detailed analysis of our data confirmed that this was not the case. Patients in which care was withdrawn early were on average older, had greater burned surfaces, greater deep burns surface, and higher Baux scores than the patients who received maximalist care (Table 4). SAPS2 score could not be calculated for 71% of these patients because of their short length of stay (death occurred on average 17 h and 46 min after their admission to ICU). These characteristics were however stable during the time of our study and, importantly, similar to the characteristics of the subgroup of patients who received maximalist care but did not survive (Table 1). This indicates that there was no bias toward withdrawal for the worst burns, but that old age or comorbidities were the deciding factors for withdrawal, in coherence with literature showing worst outcomes for elderly patients.^46,47^

**Table 4.** Comparison of early withdrawal characteristics with study’s population, medians (IQR)

Characteristic	Massive Burn Cohort	Study’s Population	Withdrawals	*P* Value
	n = 67	n = 39	n = 28	
Age (years)	40.0 (27.0–60.0)	36.0 (25.0–47.5)	65.5 (39.8–75.5)	<.001
Female (n (%))	21 (31.3)	7 (15.4)	15 (53.6)	.002
Weight (kg)	74.8 (65.0–83.0)	74.4 (68.0–83.2)	75.5 (60.0–82.0)	.377
TBSA (%)	60.0 (50.0–77.5)	65.0 (55.0–82.5)	80.0 (65.0–93.5)	.023
TBSA deep burns (%)	70.0 (60.0–89.0)	55.0 (50.0–67.5)	71.0 (55.0–87.0)	.002
Inhalation injury (n (%))	49 (73.1)	26 (64.1)	24 (85.7)	.091
SAPS2 score	NA	36.0 (32.0–45.0)	NA	NA
Modified Baux score	128.0 (111.0–154.0)	113.0 (100.5–130.0)	151.0 (135.0–170.3)	<.001

*IQR*, interquartile range; *TBSA*, total body surface area.

### European Burns Association/American Burn Association Guidelines

Our center has been following the European Burns Association (EBA)/American Burn Association (ABA) guidelines and their changes over time.^48^ Both societies’ guidelines report numerous variables associated with longer or shorter length of stay, but they make no recommendation toward aiming at shorter LICU per se. The approach was identical in our burn center where the strategies applied in the acute phase aimed primarily at the full recovery and infection control, accepting the LICU stay as essentially a consequence of these decisions.

Regarding surgical care, the EBA guidelines offer recommendations about escharotomies/fasciotomies and wound dressing only. The ABA guidelines support the use of split-thickness skin grafts, associated with dermal substitutes and/or CEAs if needed for patients with more than 40% TBSA burns.^49^

### Limitations

Despite the application of international guidelines, the external validity of this study’s results is limited by its monocentric design. Small numbers of patients and local protocols, customs, and constraints might have biased the results. By study design, only association and not causality can be tested. The chosen outcomes are objectives measures but evaluations of TBSA carry high interrater variability. Data from operative reports were cross-referenced with internal data, but collection bias might not have been entirely eliminated. The fact that the same person performed data collection and data analysis carries a risk of personal bias. A chronology bias cannot be excluded, notably in the definition of infectious episodes and in the definition of inhalation injury.

## CONCLUSIONS

The progressive amelioration in graft take rates observed over time is an encouraging sign of progress, partly influenced by changes in nutrition as well as by a reduction in infectious episodes. This however did not result in a shorter LICU stay. Mortality has not changed either, but more precise and specific outcomes focused on the quality of life and functional recovery of massively burned patients are needed.

## Notes

Funding sources: The authors did not receive any funding for this study.

Request for reprints should be sent to the corresponding author.

Author contributions: J.M., O.P., M.M.B., and W.R. designed the study. J.M. and L.W. collected the data. J.M. and M.M.B. analyzed the data. J.M., O.P., M.M.B., L.W., and W.R. wrote the manuscript. All authors contributed and approved the final version of the manuscript.
